# Persistent pathogens and wildlife reservoirs: The role of carriers in the persistence of foot-and-mouth disease in buffalo is quantified

**DOI:** 10.1126/science.abl8885

**Published:** 2021-09-30

**Authors:** Katie Hampson, Daniel Haydon

**Affiliations:** Institute of Biodiversity, Animal Health, and Comparative Medicine, University of Glasgow, Glasgow, UK

Many pathogens have an uncanny ability to persist. This enduring challenge for our understanding of infectious disease epidemiology has devastating, yet all too familiar, public health, economic, and social consequences. The conundrum is this: Explosive outbreaks caused by highly contagious pathogens rapidly deplete their susceptible supply, whereas the stuttering transmission chains of insufficiently contagious pathogens inevitably go extinct. Theory proposes that pathogen persistence runs along a spectrum. At the extremes, pathogens persist in populations large enough to maintain transmission in the troughs between epidemics, i.e., above the critical community size (CCS); or, pathogens circulate among connected but smaller populations with introductions between them maintaining circulation at larger scales, i.e., as a metapopulation (1). On page 104 of this issue, Jolles *et al*. (2) examine the persistence of foot-and-mouth disease viruses (FMDVs) in African buffalo, which are an enigma because of their persistent circulation in relatively small and isolated populations.

Highly contagious morbilliviruses are often used to illustrate the twin paradigms of pathogen persistence, with measles persisting in populations exceeding the CCS (3), and canine distemper virus persisting as a metapopulation (4). But, as our understanding of pathogen biology expands, these paradigms begin to break down. Pathogens have evolved a diverse array of life histories to increase their chance of persistence. Some, like rabies virus, West Nile virus, and coronaviruses, have a wide host range with the capability of spreading within and between species. Some can spread through previously exposed populations following waning of immunity, or through antigenic shift or drift, evading immune recognition (5). Genomic analyses have revealed how rare events prolong persistence of other pathogens—for example, flare-ups of Ebola from sexual transmission and relapsed infections (6). Indeed, with severe acute respiratory syndrome coronavirus 2 (SARS-CoV-2), it is possible to witness how rare events increase with rising infections, leading to nontrivial outcomes, such as the evolution and establishment of new variants.

Endemic FMDV, which spreads among livestock and wildlife populations, including African buffalo, remains a huge and largely neglected challenge (7). In Sub-Saharan Africa, the disease is responsible for meat and milk production losses from cattle, sheep, and goats exceeding US$2.3 billion per year (8), driving food insecurity and being consistently reported as a major concern to agropastoralists and rural smallholders (9). It also obstructs the development of lucrative export markets and economic growth more generally.

At least five FMDV serotypes circulate in Sub-Saharan Africa and each can be considered a different pathogen, although cross-reactivity means that they may interact in complicated ways (10). African buffalo are generally considered to be the natural host for FMDV, and the Southern African Territories serotypes (SATs 1 to 3) are assumed ancestral to other FMDV serotypes that exert a substantial burden of disease in livestock. Notably, SATs appear to persist in Southern African buffalo populations, with spillover into livestock mitigated by cattle vaccinations and fencing. Because FMDV is both immunizing and highly infectious, theory suggests either a large CCS or sufficient connectivity between populations—neither of which seems plausible for small buffalo herds. However, it has long been hypothesized that FMDV persistence arises from carrier infections (i.e., prolonged infection without overt disease) from which transmission may arise sporadically. Yet, direct evidence of infections from carriers to susceptible animals has only rarely been demonstrated (11).

Jolles *et al*. show that for a SAT1 strain, the carrier state extends the duration of infection, and they demonstrate how transmission from the carrier state could constitute around one-third of all potential transmission events (see the figure). Their models show that although the estimated rate of transmission from the carrier state is an order of magnitude lower than from acute infections, carriers provide a plausible explanation for observed persistence of this SAT1 strain, even in very small buffalo herds (<100 animals). More generally, their work indicates how heterogeneities in the infectious process, such as viral excretion and clearance, can cascade up to higher scales with consequences for population-level persistence. Infection experiments, like those carried out by Jolles *et al*., provide a rare window into transmission, clinical progression, and immunological responses (12). Data on individual-level viremic and immunological trajectories, both from carrier animals and their secondary infections, could reveal the mechanisms leading to the carrier state.

**Figure:**
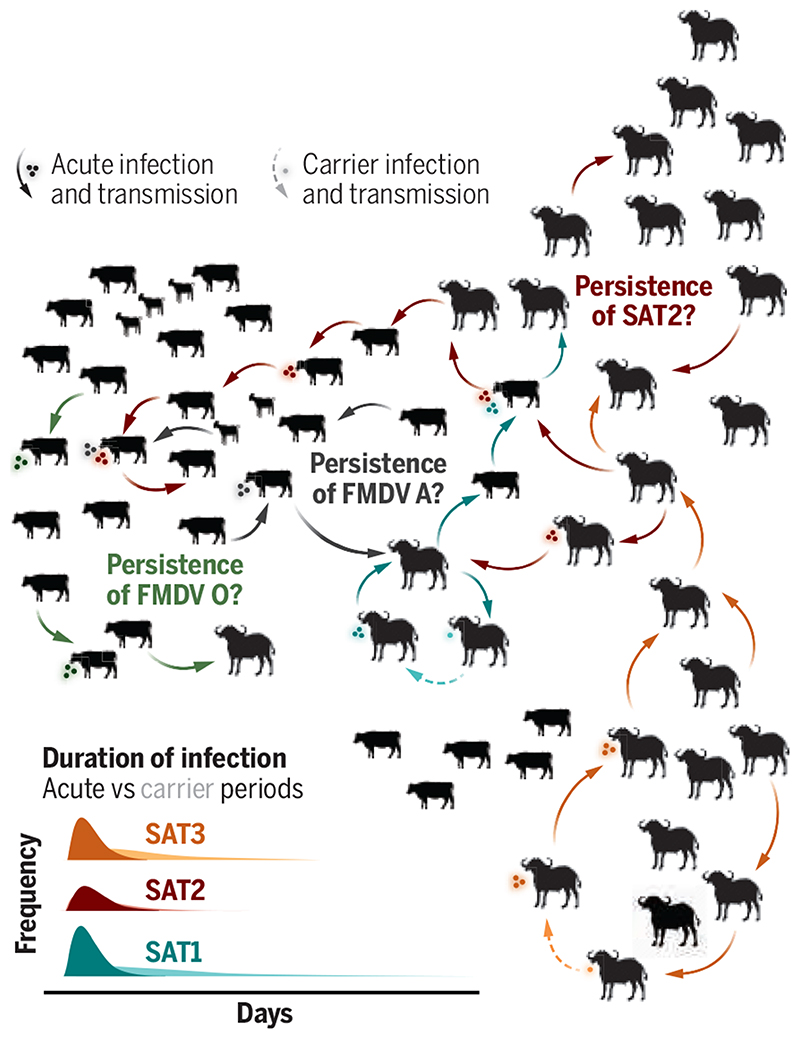
Hypothesized mechanisms of persistence Jolles *et al*. find that the life-histories of strains of Southern African Territories (SAT) serotypes of foot-and-mouth disease virus (FMDV) that co-circulate in African buffalo herds can differ considerably in their infectious period and potential for carrier transmission. They find that SAT1 carriers in buffalo are common enough to allow persistence in small, isolated herds. Additional mechanisms may be at play for other serotypes.

These SAT 1–3 strains appear to have different life histories affecting their transmission and persistence within buffalo populations, but there is some way to go before the dynamics of FMDVs in nature, particularly in livestock in which other serotypes also circulate, can be understood. Ecological processes underpinning patterns of mixing between infected and susceptible wildlife and livestock such as seasonality, age, and social system are complex and not easily captured in experimental settings.

The current practical challenge in South Africa is how to minimize the frequency of expensive FMD outbreaks in cattle. Difficulties in maintaining fencing around protected areas, as well as insufficient vaccination coverage in cattle proximal to wild buffalo populations, has resulted in a higher frequency of spillover transmissions in recent years (13). Elsewhere in Africa, serotypes O and A constitute a large proportion of the FMD burden and may circulate largely independently of wildlife (9). There is little evidence to implicate the role of carrier transmission for these serotypes. Larger-scale studies in a wider range of natural settings are required to understand the transmission dynamics of these serotypes.

Advances in the ability to study wildlife and livestock movements, access to pen-side diagnostic testing, and the affordability of large-scale genomic epidemiology should be seen as transformational opportunities to understand the complex dynamics of FMDV in Africa, as well as other important endemic and neglected viruses. The development and implementation of control policies must accelerate for countries to move along the internationally recognized Progressive Control Pathway for FMD. New scientific understanding about the persistence of these other FMDV serotypes is needed, in combination with the adoption of more progressive risk-based commodity-trade options (14), to provide practical solutions to how livestock can be productively maintained alongside wildlife and this persistent virus. Jolles *et al*. have unraveled key complexities to FMDV persistence in small buffalo populations; the time is now ripe for understanding the full spectrum of strategies for persistence of this virus across the continent.
